# Implications of Intratumor Heterogeneity on Consensus Molecular Subtype (CMS) in Colorectal Cancer

**DOI:** 10.3390/cancers13194923

**Published:** 2021-09-30

**Authors:** Saikat Chowdhury, Matan Hofree, Kangyu Lin, Dipen Maru, Scott Kopetz, John Paul Shen

**Affiliations:** 1 Department of Gastrointestinal Medical Oncology, The University of Texas MD Anderson Cancer Center, Houston, TX 77030, USA; schowdhury4@mdanderson.org (S.C.); klin1@mdanderson.org (K.L.); skopetz@mdanderson.org (S.K.); 2 Klarman Cell Observatory, Broad Institute of MIT and Harvard, Cambridge, MA 02142, USA; mhofree@broadinstitute.org; 3 Department of Biological Regulation, Weizmann Institute of Science, Rehovot 76100, Israel; 4 Department of Pathology, The University of Texas MD Anderson Cancer Center, Houston, TX 77030, USA; dmaru@mdanderson.org

**Keywords:** colorectal cancer, consensus molecular subtypes (CMS), single-cell sequencing, RNAseq, intratumor heterogeneity

## Abstract

**Simple Summary:**

Colorectal cancer (CRC) has been divided into four consensus molecular subtypes (CMS) using the unsupervised clustering of bulk transcriptomic data. The CMS, which align with known biological differences in CRC, have helped address inter-tumor heterogeneity and have been shown to be predictive of survival. However, bulk tumors are comprised of a mix of tumor cells, non-tumor stroma, and immune cells, and the relative contributions of each are not accounted for in the current CMS classification system. Here, we build an algorithm to assign CMS classification to individual cells, which we apply to single cell RNAseq data to explore the impact of intra-tumor heterogeneity on CMS. We find that the number of stromal and immune cells present has a strong influence on the bulk CMS type and that clusters of tumor epithelial cells derived from single cell RNAseq do not align with CMS.

**Abstract:**

The implications of intratumor heterogeneity on the four consensus molecular subtypes (CMS) of colorectal cancer (CRC) are not well known. Here, we use single-cell RNA sequencing (scRNASeq) to build an algorithm to assign CMS classification to individual cells, which we use to explore the distributions of CMSs in tumor and non-tumor cells. A dataset of colorectal tumors with bulk RNAseq (*n* = 3232) was used to identify CMS specific-marker gene sets. These gene sets were then applied to a discovery dataset of scRNASeq profiles (*n* = 10) to develop an algorithm for single-cell CMS (scCMS) assignment, which recapitulated the intrinsic biology of all four CMSs. The single-cell CMS assignment algorithm was used to explore the scRNASeq profiles of two prospective CRC tumors with mixed CMS via bulk sequencing. We find that every CRC tumor contains individual cells of each scCMS, as well as many individual cells that have enrichment for features of more than one scCMS (called mixed cells). scCMS4 and scCMS1 cells dominate stroma and immune cell clusters, respectively, but account for less than 3% epithelial cells. These data imply that CMS1 and CMS4 are driven by the transcriptomic contribution of immune and stromal cells, respectively, not tumor cells.

## 1. Introduction

Colorectal cancer (CRC) is the most prevalent cancer of the gastrointestinal system; nearly 900,000 people die from CRC every year worldwide [1,2,3]. In the United States alone, the Surveillance, Epidemiology, and End Results (SEER) project predicts 149,500 new cases and 52,980 deaths by CRC in the current year of 2021, making CRC the third most lethal cancer [4]. The survival rate of metastatic CRC patients beyond five years from diagnosis remains less than 20% [1], and approximately 40% of non-metastatic tumors will recur after completing surgical resection [1]. With the notable exception of BRAF V600E mutant tumors [5], other solid tumors targeted therapies (such as tyrosine kinase inhibitors, and immune therapies) have shown little benefit in CRC [6,7,8]. Existing evidence suggests that intra-tumor genetic, epigenetic, and transcriptomic heterogeneity among tumor subclones causes poor prognosis, metastasis, tumor recurrence, and drug resistivity of the CRC patients [7,9,10,11,12,13,14].

In recent clinical trials, the integration of molecular and histologic features of tumors to guide therapy has shown improvement in prognoses for CRC patients [1,15,16,17,18]. To better understand inter-tumor heterogeneity, an international consortium of colorectal cancer subtyping (CRCSC) classified CRC into four consensus molecular subtypes (CMS) based on tumor transcriptional profiles [19]. The CMS of CRC has proven beneficial for understanding CRC tumor biology and has consistently been shown to predict prognosis [19,20,21,22,23]. CMS1 is characterized by infiltrating immune cells and transcriptionally shows activation of immune response pathways. CMS2 and CMS3 are mainly enriched in the classical Wnt and metabolic pathways, respectively. Finally, CMS4 is characterized by the increased presence of mesenchymal and stromal cells, and is transcriptionally dominated by matrix remodeling, TGFβ/Integrin pathways [19,21,22]. CMS4 has been associated with poor prognosis in early-stage CRC patients [19]. However, it was shown previously that stromal cell-associated genes significantly contribute to CMS4, raising the question of whether CMS4 represents a transcriptional state of tumor cells or non-tumor stromal cells [24]. To resolve this question, the deconvolution of colorectal tumors transcriptomes into malignant and non-malignant components has been suggested to advance the understanding of the relative contributions of the tumor microenvironment in CMS classification [25].

The tumor microenvironment (TME) consisting of cancer-associated fibroblasts (CAF), infiltrating lymphocytes, macrophages, and other non-tumor cells also varies within and between CRC tissues [26,27,28,29]. Therefore the CMS framework, which solely relies on the transcriptomic profiles of bulk tumor tissues, is influenced by the aggregated transcriptome of non-malignant stromal and immune cells [30,31]. The CMS classification framework has been successfully used in in vitro assays to predict the anti-cancer drug sensitivity of CRC cell lines [32]. CMS2 cell lines have shown a strong response to anti-EGFR tyrosine kinase inhibitors or monoclonal antibodies; CMS1 cell lines have exhibited sensitivity to inhibitors of topoisomerase, mitosis, and heat shock protein 90 (HSP90); CMS4 cell lines show sensitivity to inhibitors of HSP90, HMG-CoA reductase, and alcohol dehydrogenase [32]. In addition, chemo-resistant CMS4 PDX models have shown clinical benefit from combination treatment with 5-fluorouracil and luminespib (HSP90 inhibitor) [32]. However, despite these successes, the CMS classification framework currently has limited applications in clinical decision-making for CRC patients [23,33,34,35,36,37]. A comprehensive understanding of how the heterogeneous TME and the transcriptional intratumor heterogeneity of CRC tumors—two critical biological factors that are not captured in PDX and cell lines models—impact CMS classification is still needed to advance precision oncology in CRC.

Single-cell RNA sequencing (scRNASeq) of bulk tumor samples represents a new opportunity to inspect the TME and the transcriptional intratumor heterogeneity of CRC [38,39]. Single-cell resolution allows for separation and analysis of the distinct transcriptional states of malignant and non-malignant cells of colorectal tumors, allowing for assessment of the relative contributions of different cell lineages to CMS derived from bulk transcriptomes. Although existing CMS classification algorithms, such as CMSClassifier and CMSCaller, are suitable for bulk tumor tissues, a similar assignment method for individual cells does not yet exist [19,40]. In this work, we develop for the first time a CMS assignment framework for single cells using the scRNAseq profiles from a publicly available cohort of CRC patients [41], and use the single cell CMS (scCMS) algorithm to explore intra-tumoral heterogeneity in CRC.

## 2. Materials and Methods

### 2.1. Collection of Gene Expression Profiles of the Bulk Tumor Samples

Expression-profile subsets of 5973 genes from 3232 tumor samples from the batch-effects-corrected, normalized, gene-expression profiles of the bulk tumor tissues used by the colorectal cancer subtyping consortium (CRCSC) were downloaded from the synapse data portal (https://www.synapse.org/#!Synapse:syn4961785 accessed date: 2 February 2021) [19]. All bulk tissue samples were taken from primary CRC tumors. The R library “EnsDb.Hsapiens.v86” was used to convert the ENTREZ gene ids to the corresponding gene symbols. All tumor-sample clinical and pathological information was also obtained from the same data source [19].

### 2.2. Sample Preparation and Data Processing for Single-Cell RNA Sequencing

The processed single-cell RNA sequencing (scRNAseq) profiles and corresponding metadata of ten CRC tissue samples obtained from five CRC patients (border and core regions of tumor tissue of each patient) of Commissie Medische Ethiek UZ KU Leuven/Onderzoek, Belgium, were downloaded from the National Center for Biotechnology Information Gene Expression Omnibus (GEO) database with the accession code GSE144735 [41]. This dataset (KUL3 cohort) also contained bulk-RNASeq profiles of the matched scRNASeq samples. The mRNA expression profiles of KUL3 cohort were used as the discovery dataset in this work.

Furthermore, colon adenocarcinomas of two patients (Patient #1 and Patient #2) were surgically removed at The University of Texas MD Anderson Cancer Center (UTMDACC). This study was approved by the institutional review board (IRB LAB10-0982), and written informed consents were obtained from all patients. Resected tissue was transported in ice-cold DMEM medium for further processing, and single-cell isolation and sequencing were conducted as previously described [42]. Briefly, the tumor was minced with scalpels into ~1 mm^3^ pieces, transferred into a 50 mL conical tube containing 30 mL dissociation solution, and incubated at 37 °C in a rotating mixedization oven for 15 to 60 min. The tissue suspension was subsequently passed through a 70 μm strainer and centrifuged at 450× *g* for 5 min. After the supernatant was removed, the pelleted cells were suspended in 1× MACS red blood cell (RBC) lysis buffer (MACS, Auburn, CA, USA, 130-094-183) and incubated at room temperature for 10 min. After washing with 10 mL of 4 °C DMEM, the cell pellets were re-suspended in cold phosphate-buffered saline (Sigma, St. Louis, MO, USA, D8537) + 0.04% bovine-serum-albumin solution (Ambion, Burlington, ON, Canada, AM2616) and passed through a 40 μm flowmi cell strainer (Bel-Art, Wayne, NJ, USA, h13680-0040). To make the dissociation solution, collagenase A (Sigma, Mannheim, Germany, 11088793001) was dissolved in 75% (*v*/*v*) DMEM F12/HEPES medium (Gibco, Carlsbad, CA, USA, 113300) and 25% (*v*/*v*) bovine-serum-albumin fraction V (Gibco, Carlsbad, CA, USA, 15260037) to prepare a concentration of 1 mg mL^–1^.

Single-cell capture, barcoding, and library preparation were performed by following the 10X Genomics Single-Cell Chromium 3′ (PN-120237) protocol using V3 chemistry reagents (10X Genomics). The final libraries containing barcoded single-cell transcriptomes were sequenced at 100 cycles on an S2 flowcell on the Novoseq 6000 system (Illumina, San Diego, CA, USA). Data were processed using the CASAVA 1.8.1 pipeline (Illumina), and sequence reads were converted to FASTQ files and unique-molecular-identifier read counts using the CellRanger software (10X Genomics).

The raw gene-expression matrix from the CellRanger pipeline was filtered and normalized using the Seurat v3 [43] R package, and selected according to the following criteria: >200 genes and <80% of unique-molecular-identifier counts mapping to the mitochondrial genome. After filtering, the top 2000 variable genes were selected using the “FindVariableGenes” function in Seurat for principal-component analysis. The top 10 principal components were selected to construct the shared-nearest-neighbor graph and Uniform Manifold Approximation and Projection (UMAP) embedding as implemented in Seurat. Next, single cells were identified as to cell type (e.g., epithelial-, stromal-, and immune cells) by cell-type marker expression: epithelial cells (EPCAM, KRT8, KRT18), stromal cells (COL1A1, COL1A2, COL6A1, COL6A2, VWF, PLVAP, CDH5, S100B), and immune cells (CD52, CD2, CD3D, CD3G, CD3E, CD79A, CD79B, CD14, CD16, CD68, CD83, CSF1R, FCER1G) adapted from Similie et al. [44]. Whole transcriptome profiling was also performed on the bulk tumor samples of these two patients (Patient #1 and Patient #2). Both single-cell and bulk RNASeq profiles were performed at MD Anderson Sequencing Core Facility. This dataset was used as an independent dataset for further exploration of the outcomes of discovery dataset.

### 2.3. Consensus Molecular Subtyping of the Bulk Tumor Samples

Consensus molecular subtypes (CMS) of bulk tumor samples were determined using the R library CMSClassifier, which produces the posterior probabilities of all four molecular subtypes for a given tumor sample [19]. The random forest (RF) classifier algorithm implemented in the CMSClassifier package was used to classify the four colorectal cancer (CRC) CMS subgroups: CMS1, CMS2, CMS3, and CMS4; the summation of the output of the RF classifier probabilities of all four subtypes for a given sample is equal to 1. After executing the classification algorithm, a tumor sample was assigned with one of the CMS subtypes if the posterior probability of any of the subtypes was ≥0.5; otherwise, that sample was deemed a mixed sample that contained transcriptomic signatures of multiple CRC CMSs.

### 2.4. Single-Sample Gene-Set Enrichment Analyses

The 83 gene-set database (genesets.gmt) required for single-sample, gene-set enrichment analyses (ssGSEA) was downloaded from the synapse data portal (ID: syn4961785). These were the same gene sets used by the CRCSC to characterize the transcriptomic states of each CMS [19]. Each ssGSEA was performed using ssGSEA 2.0 (https://github.com/broadinstitute/ssGSEA2.0 accessed date: 2nd February 2021) [45], and each tumor-sample gene-expression profile was normalized before using in this software. The ssGSEA 2.0 software default parameters and the database of 83 gene sets were used in all of our ssGSEA analyses.

### 2.5. Identification of CMS Specific-Marker Gene Sets

Normalized enrichment scores (NESs) of 83 gene sets from ssGSEA and the CMSs corresponding to each CRCSC-dataset tumor sample (*n* = 3232) were used to identify the gene-set characteristics (including mixed phenotype) of each CMS. Notably and compared to other CMSs (excluding the mixed group), the most significantly over- and under-represented gene sets in a molecular subtype were considered as the marker gene sets and were identified by performing hypothesis testing (using t-tests) of each NES of every molecular-subtype gene set versus those in the remaining three subtypes. Multiple hypothesis-test *p*-values were corrected by computing their false discovery rates (FDRs) where a cutoff of 0.01 was considered significant. The top five and bottom five (over and under-represented) gene sets were considered as marker gene sets for each CMS.

### 2.6. CMS Assignments to Single Cells

Gene-expression profiles obtained from scRNASeq analysis of each tumor-sample cell were normalized by converting the raw expression values into transcripts per million (TPM) scores and then log2 scaled (log2[1 + TPM]). The normalized gene-expression profile was used in ssGSEA, where each cell was considered as a distinct sample. The resulting NESs of each gene set were Z-score transformed across all single cells. The Z-score transformed NESs were further binarized to +1 if Z-score was ≥+0.5 or ≤−0.5, and to 0, otherwise. Next, the binarized enrichment scores of all CMS-specific marker gene sets identified from bulk gene expression analysis were extracted for all individual cells. Total numbers of non-zero CMS specific-marker gene sets (total = 10) corresponding to all four CMSs were counted for all single cells; given 10 gene sets per CMS the range for each was 0 to 10. Thus, single cell CMS (scCMS)-scores were calculated for all four CMSs for each single cell in a tumor. Following this, scCMS was assigned to a single cell by picking the scCMS with the highest score, if there was a tie for highest the cells were labeled as “scMixed.” Individual cells with no non-zero gene sets were assigned to the no label (scNOLBL) subtype; these contained no definite transcriptomic signature associated with any of the four CMSs. The single-cell CMS assignment algorithm is depicted in Figure S1.

### 2.7. Software and Tools

The R library CMSClassifier (ver. 1.0) was used for bulk tumor sample CMS annotation [19]. The “umap” library (ver. 0.2.2.0) was used for UMAP plotting, and the R library “pheatmap” (ver. 1.0.12) was used for plotting heatmaps. “Ward.D” algorithm implemented in “pheatmap” package was used for unsupervised clustering of NESs. The R statistical package (ver. 3.6.0) and Graphpad Prism (ver. 8.0.0) were used for basic statistical calculations. The Benjamini–Hochberg method was used for calculating FDRs accounting for multiple hypothesis correction. Repeated measures correlation and *p*-value were calculated by the R library “rmcorr” (ver. 0.4.4) [46].

## 3. Results

### 3.1. Probability Distributions of CMS Calls in Bulk Tumor Samples

**Figure 1:**
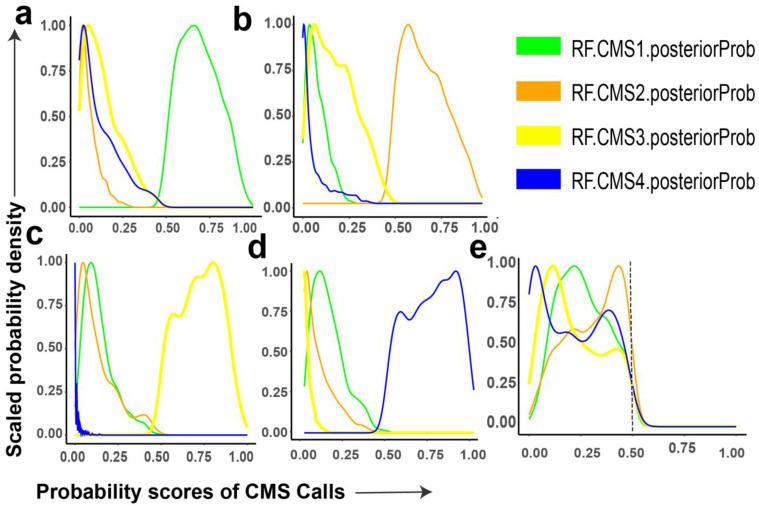
Probability densities of individual CMS calls from bulk RNAseq. (**a**) CMS1, (**b**) CMS2, (**c**) CMS3, (**d**) CMS4, (**e**) mixed group. Each CRC sample was assigned to a CMS if the probability of the CMS call was >0.50, tumors with no subtype having probability >0.50 were assigned to mixed group.

For a given tumor sample, the RF algorithm CMSClassifier provides posterior probabilities for all four CMS transcriptomic signatures [19]. We applied this method to determine the posterior probability scores of all four CMS for each bulk tumor sample in the CRCSC dataset (*n* = 3232); definitive final tumor-sample CMS call were determined in cases where there was a CMS with posterior probability value ≥0.5 (Figure 1a–d). Out of 3232 tumors, 2579 (~79%) could be definitively classified, 434, 944, 466, and 735 were classified as CMS1 (~13%), CMS2 (~30%), CMS3 (~14%), and CMS4 (~22%), respectively. The remaining 653 samples (~21%), called a mixed group, could not be definitively classified as no CMS had a posterior probability score higher than 0.5 (Figure 1e).

The average RF posterior probability score for CMS1 transcriptomic signature in CMS1 tumors was approximately 0.7, but across the hundreds of samples there was a distribution ranging from 0.5 to 1. Some samples showed probability for CMS3, CMS4, and, to a lesser extent, CMS2 (Figure 1a). Similarly, the RF posterior probability distribution for CMS2 tumors was dominated by CMS2, but with contributions from CMS3 and, to a lesser extent, CMS1, with minimal probability of CMS4 (Figure 1b). CMS3 and CMS4 appeared to be the most mutually exclusive with virtually no probability of the other in CMS3 and CMS4 tumors, respectively (Figure 1c,d). Interestingly, in the mixed group (*n* = 653, also referred to as unclassified in other CMS publications [19,32,47]), RF posterior probabilities suggested that these tumors are admixtures of multiple CMSs (Figure 1e).

### 3.2. CMS Specific-Marker Gene Sets

**Figure 2:**
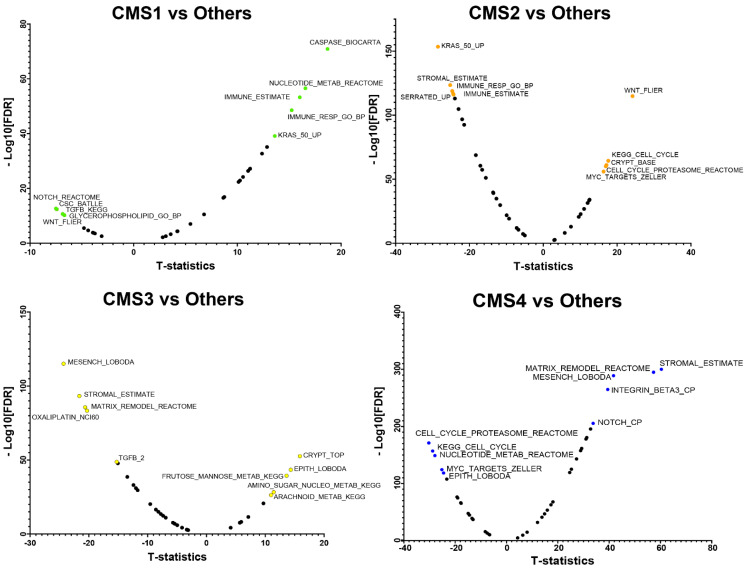
Volcano plots of CMS-specific marker gene sets (total = 83). The top five and bottom five most significant, over- and under-enriched gene sets in each CMS tumor sample are annotated in each volcano plot.

Next, we sought to identify which gene sets and/or biological pathways characterize the four CMSs and mixed subtype of CRC. We performed single sample gene set enrichment analysis (ssGSEA) on each tumor in the CRCSC dataset (*N* = 3232) using an 83 gene-set database previously used to characterize the transcriptomic states of each CMS [19]. Tumor samples were classified into five groups (four CMSs and mixed subtype), and the normalized enrichment scores (NESs) of each gene-set between one CMS versus the rest of the CMSs were compared using a *t*-test. Gene sets were ranked in descending order based on t-statistic values in each CMS, and the top five (t-stat > 0 and FDR < 0.01) and bottom five (t-stat < 0 and FDR < 0.01) gene sets (total = 10) were identified as marker gene sets of that CMS (Table S1). Positive and negative t-statistic values represent the over-enriched (active) and under-enriched (inactive) gene sets in each CMS, respectively (Figure 2). As expected, there were clear differences between CMS with most gene sets showing differential activity; however, very few gene sets/pathways (total < 10) were found to be significantly enriched (FDR < 0.01) between the mixed group vs. four CMSs (Figure S2). We did not assign gene sets as specific-markers for mixed group because there were so few significantly enriched (FDR < 0.01) gene sets relative to the four CMS.

### 3.3. CMS Represents the Transcriptomic Inter-Tumor Heterogeneity of Colorectal Cancer

**Figure 3:**
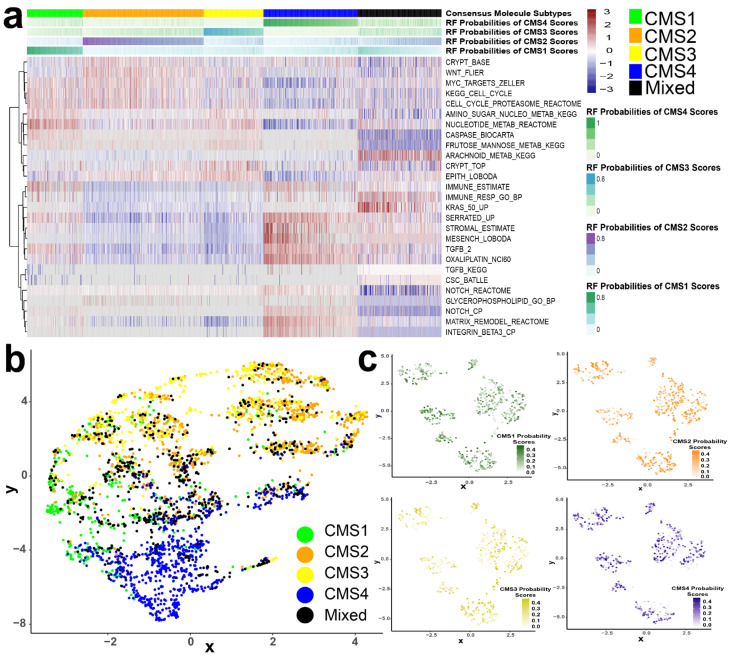
(**a**) Heatmap plot of normalized enrichment scores of 27 marker gene sets across 3232 CRC samples. (**b**) UMAP plot of all CRC patients projecting the distribution of different CMSs. (**c**) UMAP plots of tumor samples of mixed group depicting the distributions of all four CMS probability scores.

There were 27 non-redundant gene sets identified from the over-enriched (top five) and the under-enriched (bottom five) marker gene sets of each CMS group (Table S1). The NESs of these non-redundant gene sets across the 3232 CRC tissue samples were transformed to Z-scores and are shown in a heatmap plot (Figure 3a). The hierarchical clustering of gene sets indicates that CMS1-, CMS2-, CMS3-, and CMS4 tumors had homogeneous activations in their respective signature gene sets/pathways. For example, in the cluster of CMS2 tumor samples, activations of classical oncogenic signaling pathways such as Wnt, Myc, and cell cycle were observed; CMS4 tumors showed activation of stromal-, matrix-remodeling-, and mesenchymal gene sets. CMS1 tumors showed homogeneously active immune-response- and caspase pathways, and CMS3 tumors were enriched with active metabolic pathways. These results recapitulate the known ability of the CMS framework to identify inter-tumor differences in tumor biology among CRC patients [19]. In contrast, the NESs of all 27 transcriptomic signature gene sets within the mixed group of CRC samples were highly heterogeneous, indicating that these tumors had multiple active, transcriptomic signatures that did not align with a single CMS (Figure 3a). In uniform manifold approximation and projection (UMAP) of all 3232 CRC tumors, the mixed subtype did not form a distinct cluster; rather, the mixed samples aligned within the clusters of the other four CMSs (Figure 3b). This projection showed that CMS1 and CMS4 tumors tended to form distinct clusters, whereas there was not separation between CMS2 and CMS3 tumors. This observation suggests that the transcriptomic signatures of CMS1 and CMS4 tumors are more distinct than that of CMS2 and CMS3. Next, we extracted only mixed group tumors to project them on a UMAP plot and simultaneously annotated every sample with its corresponding CMS probability scores. A few subclusters were observed in mixed tumors, but none of the subclusters correlated with the probability scores of any CMS (Figure 3c). Furthermore, we did not observe any difference in survival when comparing the mixed subtype to the rest of the cohort in either early- or late-stage tumors (Figure S3). Together, these results suggest that the mixed group of CRC does not represent a distinct molecular subtype.

### 3.4. CMS Assignment for Single Cells

**Figure 4:**
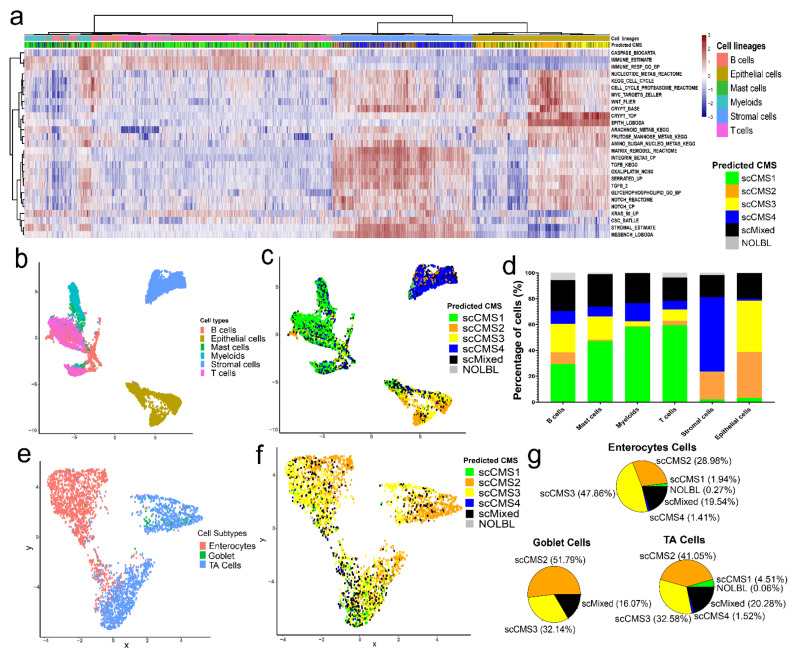
scCMS of individual cells. (**a**) Heatmap plot of normalized enrichment scores of the 27 marker gene-sets across all single tumor cells of 10 CRC samples from Lee et al. cohort. (**b**) UMAP plot of different tumor-tissue cell lineages. (**c**) UMAP plot of the four scCMS distributions and mixed transcriptomic states of the single tumor cells. (**d**) Proportions of scCMS groups in different cell lineages from Lee et al. scRNASeq dataset. (**e**) The UMAP plot of the epithelial-cell subtypes. (**f**) scCMS distributions within the epithelial-cell cluster. (**g**) Proportions of scCMSs in the populations of enterocytes and goblet- and transit-amplifying (TA) cells.

A public dataset containing single-cell transcriptomic profiles for 10 tumors from 5 CRC patients (obtained from core and border regions of each tumor tissue) was used as a discovery dataset [41]. Single-cell gene-expression profiles of 13,627 single cells were obtained from 10 tumor samples. The NESs of the 27 marker gene sets in each tumor-sample cell were pooled together into a single data matrix of 27 rows (gene sets) and 13,627 columns (single cells). The single cells obtained in the scRNASeq profile of each tumor sample were pre-classified in the discovery dataset as B-, mast-, myeloid-, T-, epithelial-, and stromal cells [41], with 2272 B-cells (16.7%), 172 mast cells (1.3%), 1158 myeloid cells (8.5%), 3532 T-cells (25.9%), 3192 epithelial cells (23.4%), and 3301 stromal cells (24.2%). Unsupervised hierarchical clustering of the data matrix clearly segregated immune cells (B-, mast-, myeloid-, and T-cells), epithelial cells, and stromal cells into three distinct clusters (Figure 4a). The higher than expected percentage of immune cells is likely due to the fact that these cells preferentially survive the dissociation process [48,49,50,51]. Distinct clusters of immune-, epithelial-, and stromal cells were also seen in a UMAP (Figure 4b) plot. The NESs of the 27 gene sets from each cell were used to construct a UMAP plot. As expected, the NESs of the 27 CMS-specific marker gene sets from individual tumor-cell clustered according to their cell lineage (i.e., immune, epithelial, and stroma). We did not find any batch effect or variations of NESs of the marker gene sets among different tumor samples of discovery dataset (Lee et al. cohort) in UMAP plot (Figure S4a). Additionally we evaluated expression of the known T cell markers PD1 [52] and FoxP3 [53], monocyte marker CD163 [54] and macrophage marker PD-L1 [55] and confirmed that expression of these genes was localized to T cells and myeloid cells, respectively (Figure S4b). Individual cells from all tumor samples were homogeneously distributed across all three cell lineages clusters. This result suggests that single-cell RNASeq counts of the discovery dataset were normalized, and variations among different tumor samples were minimized [41]. However, we could see some clustering by sample within epithelial cells subset, which is an expected result given inter-tumoral heterogeneity of transcription.

Next, we assigned scCMSs to individual cells (total cells = 13,627) for each tumor sample of discovery dataset using our newly developed single-cell CMS assignment algorithm. In this algorithm, instead of using mRNA expression profiles, the normalized enrichment scores of CMS specific-marker gene sets (Table S1) of individual cells were utilized to assign one of the four scCMS to a single cell. We applied the scCMS assignment algorithm on every single cell of the tumor tissue irrespective of cell type or lineage. To determine the scCMS of a given single cell using this algorithm, we first converted the normalized enrichment scores of the gene sets to Z-scores across all cells. We then transformed the Z-scores to binary scales of either one or zero. The total number of non-zero CMS specific-marker gene sets was computed for every single cell and labeled scCMS score (see methods). Heatmap plots of NESs of marker gene sets recapitulated several aspects of known biology; immune lineage cells had active immune-signature gene sets, stromal cells had active matrix-remodeling- and stromal-cell signature gene sets. Greater heterogeneity was seen in the epithelial lineage with one cluster of cells strongly enriched for Wnt, crypt and epithelial gene sets, and a second cluster without a defining highly active gene set (Figure 4a).

### 3.5. Distributions of scCMSs Vary among Cell Lineages

The single-cell CMS assignment algorithm predicted 3689 (27.0%) scCMS1; 2169 (15.9%) scCMS2; 2161 (15.9%) scCMS3; 2594 (19.0%) scCMS4; 2694 (19.8%) scMixed-transcriptome state; and 320 (2.4%) unclassified or NOLBL cells out of a total of 13,627 single cells in the discovery dataset. Projecting these data into two-dimensional UMAP plots showed a clear association between cell lineage and scCMS (Figure 4b,c). As expected, scCMS1 cells were highly enriched in immune cells, and the large number of immune cells (52.4%) in these samples explains why scCMS1 accounted for the largest number of cells overall (Figure 4d). The stromal-cell lineage cluster was highly enriched with scCMS4 cells, whereas the epithelial-cell lineage cluster had tumor cells of both scCMS2 and scCMS3 as major molecular subtypes. The algorithm also predicted a substantial proportion (19.8%) of mixed tumor cells (scMixed) with active transcriptomic signatures of multiple CMSs. A small number of cells showed activation of none of the hallmark CMS signatures which were identified as NOLBL cells (2.4%).

### 3.6. scCMSs Are Associated with Transcriptomic Intra-Tumor Heterogeneity in CRC Tissues

Cells with scCMS1 transcriptomic signature were present in significant numbers in the CRC-tissue immune-cell lineages (29.3% in B-cells, 47.1% in mast cells, 58.2% in myeloid cells, and 59.5% in T-cells) (Figure 4d). Large proportions (57.8%) of scCMS4 cells were found in the stromal-cell populations. The epithelial-cell lineage population mostly contained scCMS2 (35.6%) and scCMS3 (39.7%) tumor cells with only rare scCMS1 (3.2%) and scCMS4 (1.44%) tumor cells in that population. The single tumor cells with mixed-CMS transcriptomic signatures were substantially present in all cell-lineage populations and sub-types (Figure 4d). As a whole, scCMS assignment was strongly associated with distributions of different cell lineages (chi-squared statistic = 10412, df = 25, *p*-value < 0.001).

**Figure 5:**
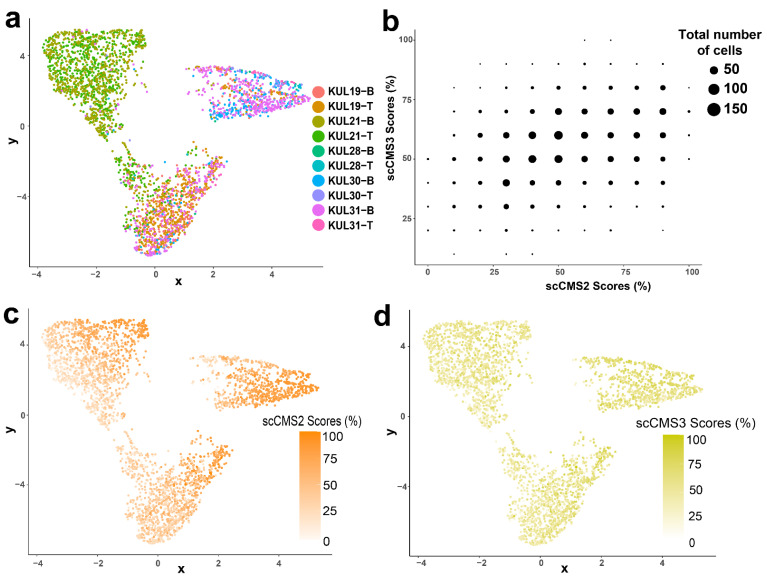
UMAP plots of scCMS2 and scCMS3 scores in epithelial cells clusters. Same UMAP projection as Figure 4e,f but now colored by (**a**) tumor sample ids. (**b**) Scatter plot showing scCMS2 and scCMS2 scores of each epithelial cells. UMAP plots of (**c**) scCMS2 and (**d**) scCMS3 scores of individual epithelial cells.

Epithelial cells could be further subclassified into enterocytes (46.8%), transit-amplifying (TA) cells (51.4%) as well as rare goblet cells (1.8%) [41]. Unsupervised clustering of the epithelial cell population showed three distinct clusters—the first largely enterocytes by themselves, the second a mix of enterocyte and TA cells, and the third largely TA cells but also including the goblet cells (Figure 4e). Epithelial cells of scCMS2, scCMS3, and mixed subtype were present in all three clusters in roughly equal proportions (Figure 4f); scCMS3 was the most frequent subtype amongst enterocytes (47.9%), while scCMS2 was most frequent in TA (41.1%) and (51.8%) Goblet cells. Notably, scCMS1 and scCMS4 cells were rare in both enterocytes and TA cells, and absent from goblet cells (Figure 4g). The fact that epithelial cells clustered first by sublineage rather than scCMS suggests the transcriptomic differences between sublineage are greater than those between scCMS2 and scCMS3. Interestingly, the epithelial cells do cluster by patient (Figure 5a) indicating that NES of the 27 CMS-specific-marker gene sets can capture important inter-tumor heterogeneity. However, the transcriptional states defined by scCMS2 and scCMS3 do not separate the tumor cells in a meaningful way. Essentially, all epithelial cells showed enrichment of some scCMS2 and scCMS3 gene sets, and these enrichment scores were roughly correlated (Figure 5b). Plotting in two dimensions as UMAP also did not reveal any separation of scCMS2 and scCMS3 scores (Figure 5c,d). These data suggest that applying transcriptomic signatures derived from bulk transcriptomic sequencing to single cells does not capture the heterogeneity of tumor intrinsic epithelial cells.

**Figure 6:**
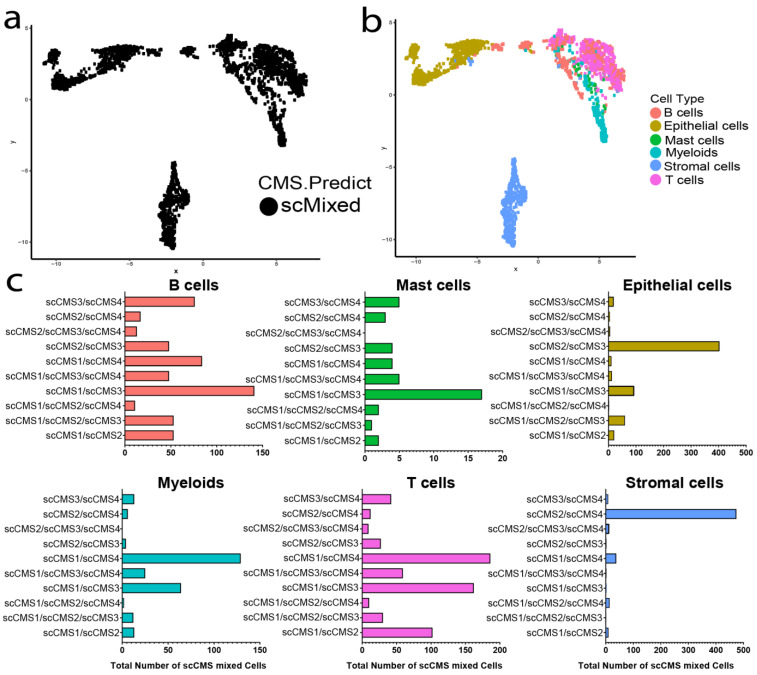
Individual cells with mixed CMS. (**a**) UMAP plot of single tumor cells with mixed transcriptomic states. (**b**) Cell-lineage/cell-type distributions in cells with mixed transcriptomic states. (**c**) Total scCMS-mixed cells within each colorectal-cancer-tissue cell lineage.

Further study of single cells with mixed transcriptomic signatures revealed heterogeneous gene-expression patterns in scMixed cells. scMixed cells produced three distinct clusters separated by cell lineage (Figure 6a,b). Just as scCMS1 was the dominant subtype in the immune-cell cluster, the majority of mixed cells in the same cluster had contribution from scCMS1. Combined with the 49.3% of immune cells that were outright scCMS1, 81.3% of immune cells showed some scCMS1 expression signature in total. In the epithelial-cell cluster, the mixed cells mainly contained transcriptomic signatures of scCMS2 & scCMS3, the dominant subtypes in that lineage. Similarly, the stromal-cell cluster of scMixed cells was dominated by scCMS2 with scCMS4, and rare combinations of scCMS4 and other scCMS were also present (Figure 6c).

### 3.7. Frequency Distributions of Single Cells CMSs Do Not Correlate with CMS of Matched Bulk Tumor

Both bulk and single-cell RNASeq profiles were available for all tumor samples (*N* = 10) in the discovery dataset [41], which allowed for a comparison of scCMS frequency in scRNASeq profile with the RF probability score for each CMS in bulk RNASeq. We computed the cell frequencies of all four scCMSs, scMixed, and unclassified/no-labeled (NOLBL) cells in each scRNASeq profile of all ten tumor samples; and separately calculated posterior probability scores of all four CMSs of each bulk tumor sample using the conventional random forest (RF) classifier of CMS (Table S2) [19]. In the RF classifier of bulk tissue samples, the CMS with the highest posterior probability score (>0.5) was considered the predicted CMS of that tumor sample [19,41]. The predicted CMSs of the bulk tumor samples (*n* = 10) were concordant with the CMSs reported in the previous study of the same tumor samples [41]. However, we found that there was not a correlation between the posterior probability from bulk CMS assignment and percentage of cells with given scCMS (Repeated measures correlation = −0.26, *p*-value = 0.15). However, with only ten samples from five tumors, this cohort was not powered to identify correlations in scCMS percentage with specific bulk CMS subtypes. In general, there was less variation in scCMS percentage across tumors relative to variation in posterior probability scores (Table S2). Given the strong association of cell lineage with scCMS, the discrepancy between CMS predictions in bulk tissue samples and observed frequencies of the single cells is most likely due to selective loss and/or enrichment of particular cell types during tumor tissue disaggregation [50,51,56,57,58,59].

### 3.8. Exploring the Associations of CMS and Intra-Tumor Heterogeneity in an Independent Dataset

**Figure 7:**
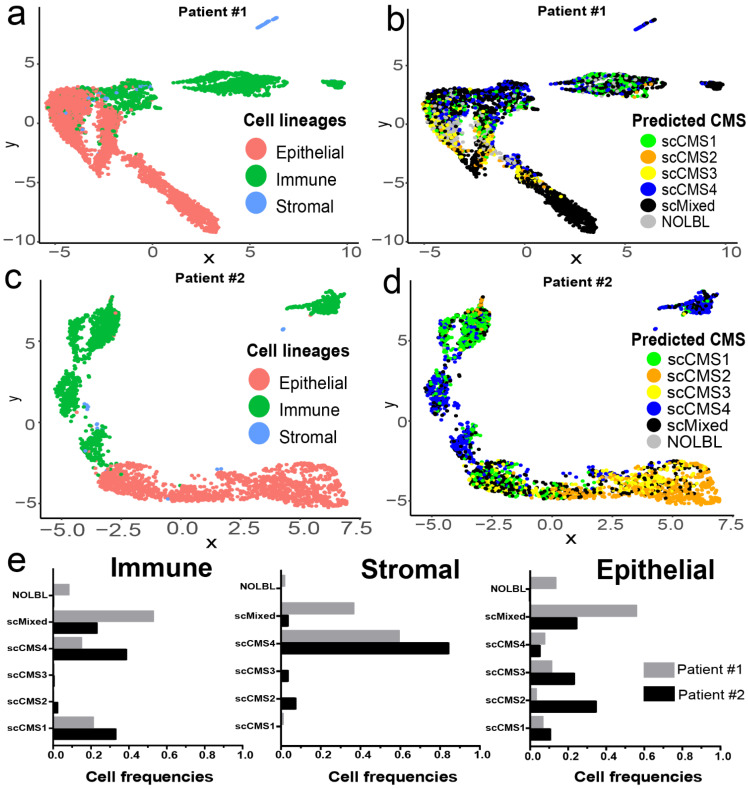
scCMSs from two CRC with mixed subtype from bulk transcriptome. UMAP plots of different single-cell types and scCMS predictions in (**a**,**b**) Patient #1 and (**c**,**d**) Patient #2 scRNAseq profiles, respectively. UMAP plots of single tumor cells were plotted using the normalized enrichment scores of the 27 marker gene sets (Figure 3) obtained from ssGSEA. (**e**) Distributions of scCMSs in immune-, epithelial-, and stromal cell populations in patient #1 and patient #2.

To further explore single-cell CMS assignments we generated single-cell transcriptomic profiles for two CRC patients (patient #1 and patient #2) with mixed CMS from bulk classifier. UMAP plots of all single cells from these tumors showed three distinct clusters corresponding to immune-, stromal-, and epithelial-cell lineages (Figure 7a,c). The scCMS distributions in patient #1 showed clear separations with immune- (primarily enriched with scCMS1 and scCMS4), stromal- (predominantly scCMS4), and epithelial-cell (populated with scCMS3 and small fractions of scCMS1, scCMS2, and scCMS4) clusters (Figure 7b,e). In contrast, higher proportions of scMixed cells were present in all three cell lineages of patient #2, and the separation of immune and epithelial cells was less distinct (Figure 7d,e). However, both tumors contained immune-cell scCMS1 and stromal-cell scCMS4 and epithelial-cell clusters enriched with scCMS2 or scCMS3 cells similar to the discovery cohort. The percentage of scMixed cells and their distribution among the three lineages was also roughly similar to the discovery cohort (Table S2). These finding suggest that tumors assigned mixed subtype from bulk CMS are admixtures of cells in similar transcriptomic state to other CMS, rather than containing cells with a unique hybrid transcriptional state.

## 4. Discussion

The CMS classification system for CRC has proven effective in understanding disease prognosis and tumor biology, with distinct gene sets and oncogenic pathways significantly associated with each of the four different subtypes (CMSs) [19,20,22,60]. However, having been developed using bulk transcriptomic data, there is now increasing recognition that there are distinct contributions to bulk CMS from both tumor and non-tumor cells and that CMS may not be adequate to capture intra-tumor heterogeneity [24,61,62,63]. Specifically, the classification of CMS4 tumors has been identified as primarily influenced by genes expressed in cancer-associated fibroblasts (CAFs) and other stromal cell subtypes [64,65,66,67]. Recognizing this stromal influence, newer CMS classification algorithms have been developed to minimizing the effect of CAF expressed genes, which is of particular importance in cell line and organoid CRC models [40,68,69,70,71]. Similarly, CMS1 is known to be associated with greater infiltration of immune cells [19,72]. Additionally, even as CMS testing moves into the clinic as a CLIA certified assay, the fact that 10–20% of tumors do not have a dominant probability amongst the four CMS and remain classified as mixed remains an unresolved issue with the current CMS categorization system [73]. Greater intra-tumor heterogeneity has been independently associated with worse survival [61]; however, with only bulk transcriptomics, it is not possible to determine with certainty if these samples with mixed CMS probability represent mixtures of cells with transcriptomic state similar to one of the known CMS, if these tumors consist of individual cells in a hybrid transcriptomic state, or if there is contribution from each scenario.

The technical ability to perform single-cell transcriptome measurements now provides the ability to explore intra-tumor heterogeneity with previously unprecedented resolution [74]. Prior scRNASeq studies in CRC have explored intercellular interaction networks between cancer and immune or stromal cells populations [41] and identified subclonal structures in tumor cells [66]. In order to better understand intra-tumor heterogeneity in the context of CMS, we sought to develop the first method to assign scCMS to individual cells using scRNASeq profiles of CRC tumors. Single cell CMS assignment has been challenging due to the intrinsic differences of bulk and single cell RNA sequencing. Bulk RNAseq will generally produce tens to hundreds of million reads per tumor, and a non-zero expression values for over 18,000 genes. In contrast, scRNASeq generated with the commonly used droplet-based technologies will generate non-zero expression for only ~3000 genes [75], preventing a bulk CMS classifier from being applied directly to single cell data. To mitigate the issue of sparsity in the single cell data we have developed a new single-cell CMS assignment algorithm that first converts gene expression profiles into gene set enrichment scores (Figure 4). Similar to other network-based approaches, using normalized enrichment scores (NES) (a measurement of the activity of gene sets or oncogenic pathways) to aggregate the expression of multiple genes minimized the effects of feature drop-outs and improved the signal-to-noise ratio in scRNASeq profile [76,77]. The 27 non-redundant active (positively enriched) and inactive (negatively enriched) marker gene sets that we use to make CMS assignments recapitulate the known biological differences in CMS, providing confidence in this approach (Table S1, Figure 3). It is also important to note that the process of disaggregating a solid tumor into a single cell suspension will intrinsically bias the cell proportions observed in single cell data [50,51,56,57,58]; this likely explains the lack of correlation between CMS probability of bulk tissue and proportion of scCMS cells, and the overall high percentage of immune cells seen in single cell data. Similar studies comparing bulk RNAseq to pseudo-bulk RNAseq (aggregation of single cell RNAseq for a tumor) have shown similar discrepancies, suggesting that this issue biased cell populations from disaggregation is common to all solid tumors [59,78].

Applying scCMS classification to individual cells we find a striking association between cell lineage and scCMS. Immune cells are predominantly assigned to scCMS1 and stromal cells are predominantly assigned to scCMS4 with a small portion assigned to scCMS2 or mixed. However, the lineage association is most striking in epithelial cells, where we observe a near complete absence of scCMS1 or scCMS4 cells (Figure 4f,g). Even the mixed cells in the epithelial compartment are almost entirely mixes of scCMS2 and scCMS3 (Figure 6c). On average epithelial cells had binarized NES scores of 2.5 and 1.4 for scCMS1 and scCMS4, respectively, which indicates minimal contribution (Figure S5). Taken as a whole, these data indicate that scCMS1 and scCMS4 are not tumor intrinsic gene expression signatures, but rather are derived from immune and stromal cells. This conclusion is also supported by the bulk tissue CMS probability scores from the much larger number of bulk tumors, where CMS1 and CMS4 tumors have significant probability for CMS2 and CMS3 in addition to the dominant probability (Figure 1). In bulk CMS1 or CMS4 tumors, the gene expression contribution of immune or stromal cells hides potential differences in the transcriptional state of the tumor cells. More generally, while the transcriptional signatures from bulk transcriptomic sequencing do separate cells by lineage, within the epithelial lineage the scCMS classifications do not capture the major transcriptional differences between individual cells. Although clustering is seen, suggesting that different single cell transcriptional states exists, these clusters did not separate between scCMS2 and scCMS3. Of the 3192 epithelial cells, including those from CMS1 and CMS4 tumors, 3178 (99.5%) had enrichment of at least one CMS2 gene set, and all 3192 (100%) had enrichment of at least one CMS3 gene set. These results support the conclusion that CMS2 and CMS3 are tumor intrinsic signatures, but also suggests that there is overlap between two CMS2 and CMS3. This finding is also consistent with genomic studies of CRC which show nearly universal alteration in Wnt [79], and the poor alignment of bulk CMS with somatic mutations [20]. Regarding tumors classified as mixed from bulk RNAseq we find both a mixture of cells that can be assigned to one of the four CMS as well as individual cells in a mixed transcriptional state. The UMAP projection of these tumors show distinct subclusters within the epithelial compartment, with some alignment of the clusters and single cell CMS. These data indicate that bulk mixed CMS tumors may have greater heterogeneity between tumor cells, which may contribute to recent finding associating these tumors with poor prognosis in localized CRC [61].

The observed association of scCMS with cell lineage has several important implications for the molecular subtyping of CRC. It is now widely recognized that the degree of immune infiltration and stromal contribution to the tumor microenvironment influences CRC tumor biology, prognosis, and response to therapy [28,29,65,72]. However, recognizing that two of the four CMS are derived from non-tumor transcriptomes can help explain the complicated and sometimes conflicting association of CMS with response to therapy [36,80]. Recognizing that the original CMS was only validating in primary colon tumors, separating tumor intrinsic expression from TME will be important to expand CMS classification to tissue samples taken from the liver and other metastatic sites. Additionally, recognizing the spatial heterogeneity of large CRC tumors (thus separating the tumor from TME) should also minimize instances where a single tumor is classified differently based on the region sampled [24]. As the number of CRC tumors with single cell transcriptomic profiling continues to increase, it may be possible in the future to define the transcriptomic states of CRC tumor cells specifically, enabling a tumor to be described by multiple complementary features: tumor intrinsic, immune infiltration, and stromal reaction. Separating these components will allow for the observation of interactions between the cell types and provide a better framework to understand how tumors respond when treated with a combination of cytotoxic and targeted chemotherapies. It is also anticipated that subtypes derived from single cell data would better align with tumor somatic mutation profiles, which has been a prior criticism of the CMS. Given the cost and sample preparation issues of single cell transcriptomics it may never be possible to generate such data routinely in the clinic. However, recently significant advances have been made in the deconvolution of bulk transcriptomic data into tumor, immune and stromal contributions [61]. Combining such methods with single cell derived transcriptomic subtypes may yield a CRC tumor classification better equipped to account for the intra-tumor heterogeneity seen in these tumors.

It should be taken into account that these transcriptomic profiles represent a single snapshot of the tumor, which is known to evolve over time. Selective pressure exerted by chemotherapy is known to alter the landscape of interactions between tumor and the microenvironment. Future studies that involve longitudinal data, that is serial sampling of the same tumor over time, are needed to understand the evolution of intra-tumor heterogeneity.

This study has multiple limitations, notably its small sample size and single time point measurement of each tumor. It is therefore not possible to examine dynamic changes of scCMS. Also, the present study does not discuss the predictive potential of scCMS profiling of CRC patients as it was conducted on a small number of patients with limited clinical information. In future work, new gene signatures could be identified from scRNASeq profiles and correlated with early relapse, tumor metastases, or therapy resistance. Spatial single-cell transcriptomic profile and information of cell to cell communication networks could be integrated with scCMS framework to extract clusters of tumor cells with novel gene expressions signatures aligned with clinical and molecular properties of CRC tissues.

## 5. Conclusions

We identify a strong association between cell lineage and single-cell CMS (scCMS). Epithelial cells, which include all of the tumor cells, are exclusively classified as scCMS2 or scCMS3, indicating that scCMS1 and scCMS4 are in fact signatures of the TME and not the intrinsic tumor transcriptome. To better account for the observed intra-tumor heterogeneity in CRC tumors, future classifications systems may benefit from defining transcriptional states from single cell, rather than bulk data.

## Data Availability

The data presented in this study are available on request from the corresponding author.

## Supplementary Materials

The following are available online at https://www.mdpi.com/article/10.3390/cancers13194923/s1, Figure S1: Flow chart of single cell CMS (scCMS) classification, Figure S2: Gene sets enriched in the mixed group of CRC tissues, Figure S3: KM plots of relapse-free survival probabilities of combined CMS1/2/3/4 vs. mixed group in CRCSC dataset, Figure S4: UMAP plot of discovery CRC single-cell dataset, Figure S5: UMAP plots of specific CMS signal in the discovery dataset, Table S1: Marker gene sets for each consensus molecular subgroup, Table S2: Frequencies of scCMS and probabilities of bulk CMS in discovery and exploratory datasets.
