# Supplementary material for: Implications of Intratumor Heterogeneity on Consensus Molecular Subtype (CMS) in Colorectal Cancer
Source: Cancers (Basel). 2021 Sep 30;13(19):4923. doi: 10.3390/cancers13194923 (PMC8507736; doi:10.3390/cancers13194923)

Supplementary Information

Implications of intratumor heterogeneity on consensus molecular subtype (CMS) in colorectal cancer

Saikat Chowdhury[1], Matan Hofree[2], Kangyu Lin[1], Dipen Maru[3], Scott Kopetz[1], John Paul Shen[1*]

[1] Department of Gastrointestinal Medical Oncology, The University of Texas MD Anderson Cancer Center, Houston, Texas 77030, USA

[2] Klarman Cell Observatory, Broad Institute of MIT and Harvard, Cambridge, Massachusetts 02142, USA

[3] Department of Pathology, The University of Texas MD Anderson Cancer Center, Houston, Texas 77030, USA

* Correspondence: jshen8@mdanderson.org

Supplementary Tables:

Table S1: Marker gene sets for each consensus molecular subgroup

	CMS1	CMS2	CMS3	CMS4
Top 5 (over-enriched)				
Pathways/ Gene sets	1. Caspase<br>2. Nucleotide metabolism<br>3. Immune response<br>4. KRAS<br>5. Immune estimate	1. Notch<br>2. TGFβ<br>3. Glycerophospholipid<br>4. Wnt<br>5. Crypt-base	1. Fructose-mannose<br>2. Amino-sugar-nucleotide metabolic<br>3. Arachnoid metabolic<br>4. Crypt-top<br>5. Epithelial-cell signature	1. Integrin-β<br>2. Notch<br>3. Stromal estimate<br>4. Matrix remodeling<br>5. Mesenchymal-cell signature
Bottom 5 (under-enriched)				
Pathways/ Gene sets	1. Notch<br>2. TGFβ<br>3. Glycerophospholipid<br>4. Wnt<br>5. cancer stem-cells	1. KRAS<br>2. Immune response<br>3. Active serrated<br>4. Stromal estimate<br>5. Immune estimate	1. TGFβ<br>2. Mesenchymal-cell Signature<br>3. Stromal estmate<br>4. Matrix remodeling<br>5. Oxaliplatin drug-induced	1. Nucleotide-metabolism<br>2. MYC<br>3. Proteasome-mediated cell cycle (Reactome)<br>4. Cell-cycle pathway (KEGG)<br>5. Epithelial cell-signature

Abbreviations: TGF-β = transforming growth factor-β

Table S2: Frequencies of scCMS and probabilities of bulk CMS in discovery and exploratory datasets

scRNASeq Profile	scCMS1 Freq.	CMS1 Prob.	scCMS2 Freq.	CMS2 Prob.	scCMS3 Freq.	CMS3 Prob	scCMS4 Freq.	CMS4 Prob.	scMixed Freq.	NOLBL Freq.	RF.nearestCMS	RF.predictedCMS
Discovery dataset												
KUL19-T	0.34	0.11	0.15	0.02	0.13	0.01	0.13	0.86	0.21	0.04	CMS4	CMS4
KUL19-B	0.31	0.15	0.16	0.53	0.15	0.3	0.12	0.02	0.22	0.04	CMS2	CMS2
KUL21-T	0.22	0.65	0.15	0.03	0.18	0.11	0.25	0.21	0.19	0.01	CMS1	CMS1
KUL21-B	0.22	0.63	0.16	0.02	0.21	0.12	0.23	0.23	0.18	0	CMS1	CMS1
KUL28-T	0.21	0.11	0.20	0.75	0.14	0.12	0.24	0.02	0.16	0.05	CMS2	CMS2
KUL28-B	0.15	0.04	0.14	0.52	0.26	0.44	0.27	0	0.17	0.01	CMS2	CMS2
KUL30-T	0.18	0.11	0.14	0.67	0.16	0.08	0.29	0.14	0.20	0.03	CMS2	CMS2
KUL30-B	0.23	0.06	0.14	0.73	0.14	0.17	0.29	0.04	0.19	0.01	CMS2	CMS2
KUL31-T	0.17	0.09	0.26	0.25	0.12	0.65	0.17	0.01	0.27	0.01	CMS3	CMS3
KUL31-B	0.29	0.06	0.21	0.28	0.13	0.65	0.20	0.01	0.16	0.01	CMS3	CMS3
Exploratory dataset												
Patient #1	0.22	0.39	0.20	0.06	0.13	0.41	0.22	0.14	0.24	0	CMS3	Mixed/Undermined
Patient #2	0.21	0.20	0.18	0.24	0.14	0.09	0.22	0.47	0.24	0.01	CMS4	Mixed/Undermined

Freq. = Frequency; Prob. = Probability; RF = Random forest

Supplementary Figures:

Figure S1: Flow chart of single cell CMS (scCMS) classification

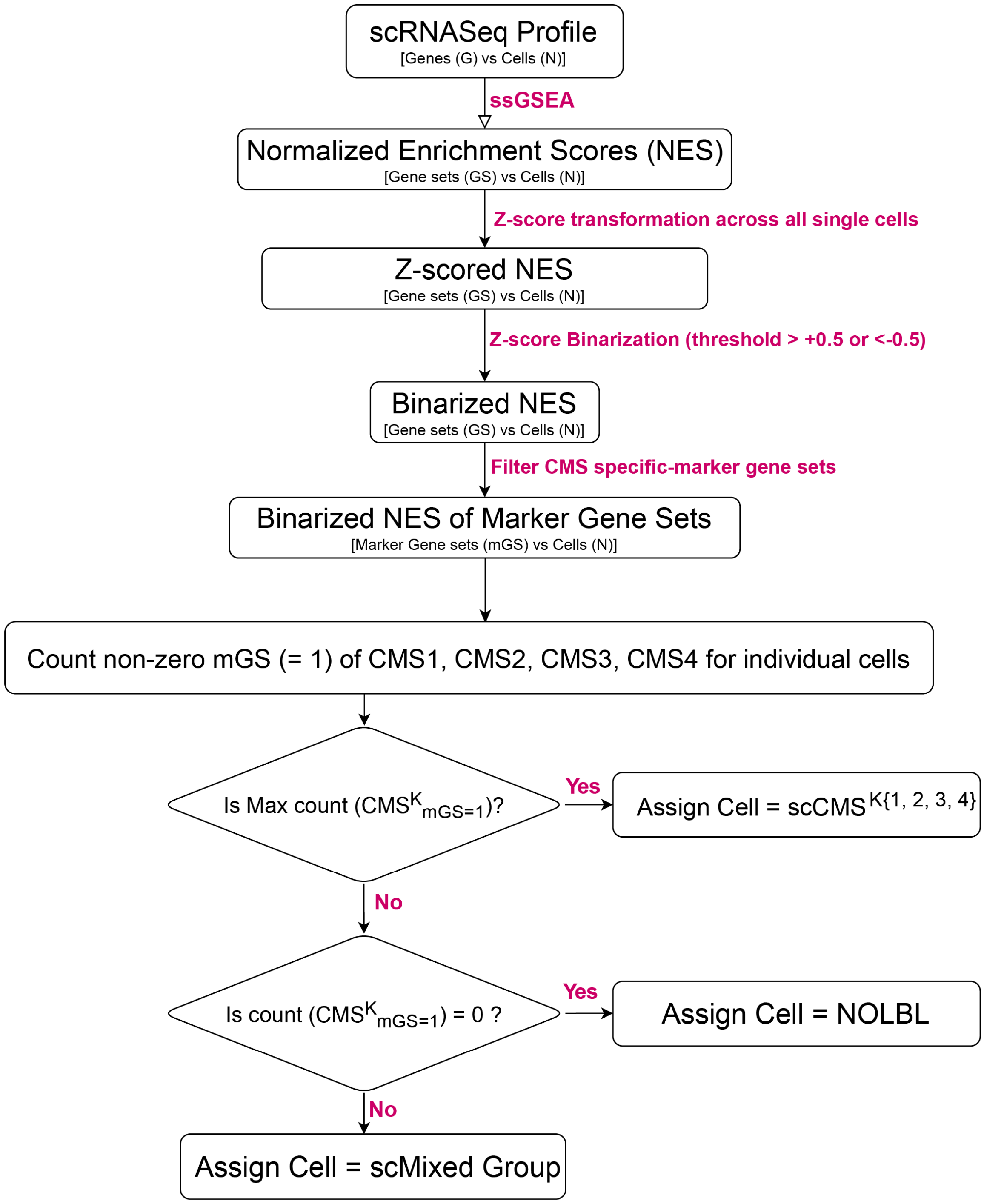

Figure S2: Gene sets enriched in the mixed group of CRC tissues

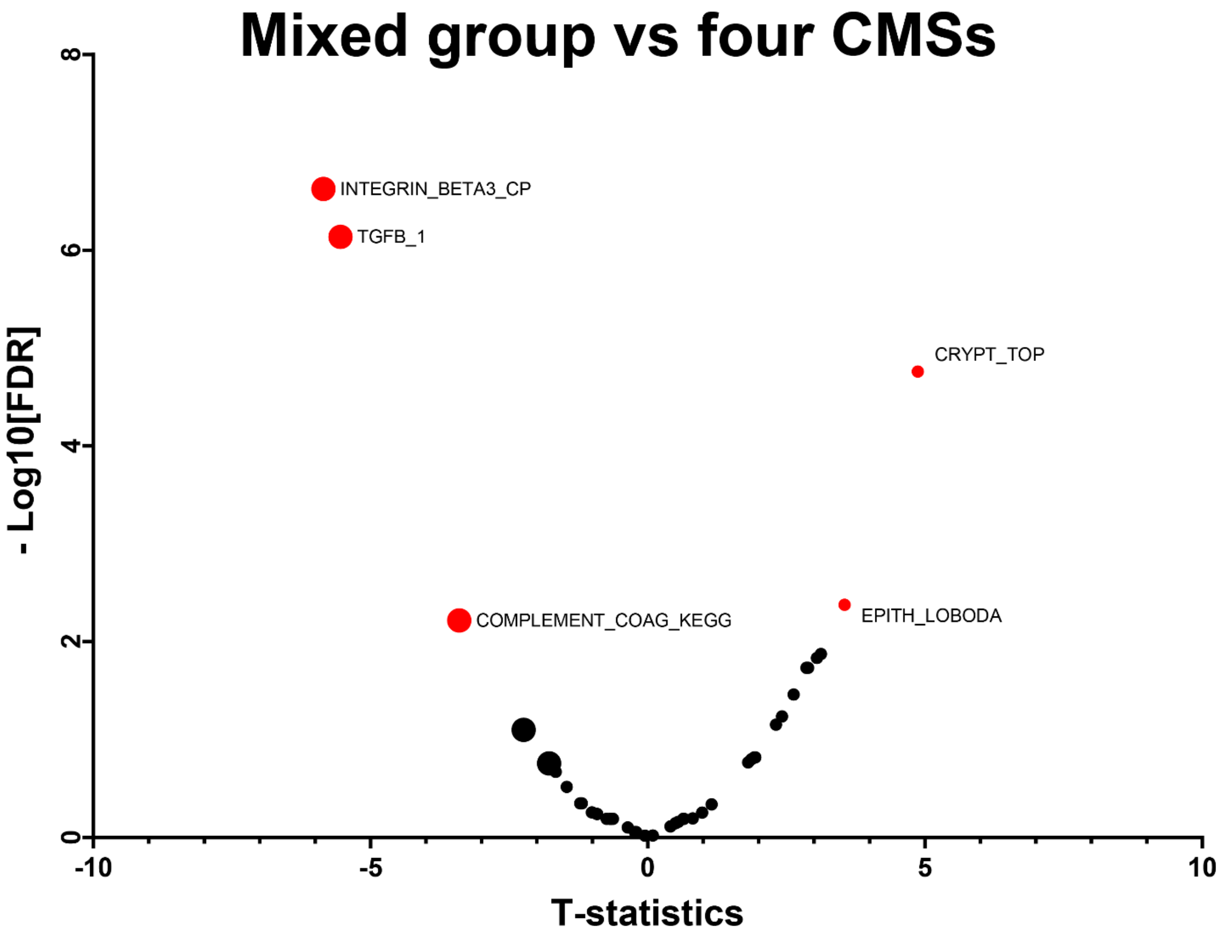

Figure S3: KM plots of relapse-free survival probabilities of combined CMS1/2/3/4 vs. mixed group in CRCSC dataset. (a) Overall survival in stage IV patients, (b) relapse free survival in stage IV patients, (c) overall survival in early stage patients, (d) relapse-free survival in early stage patients.

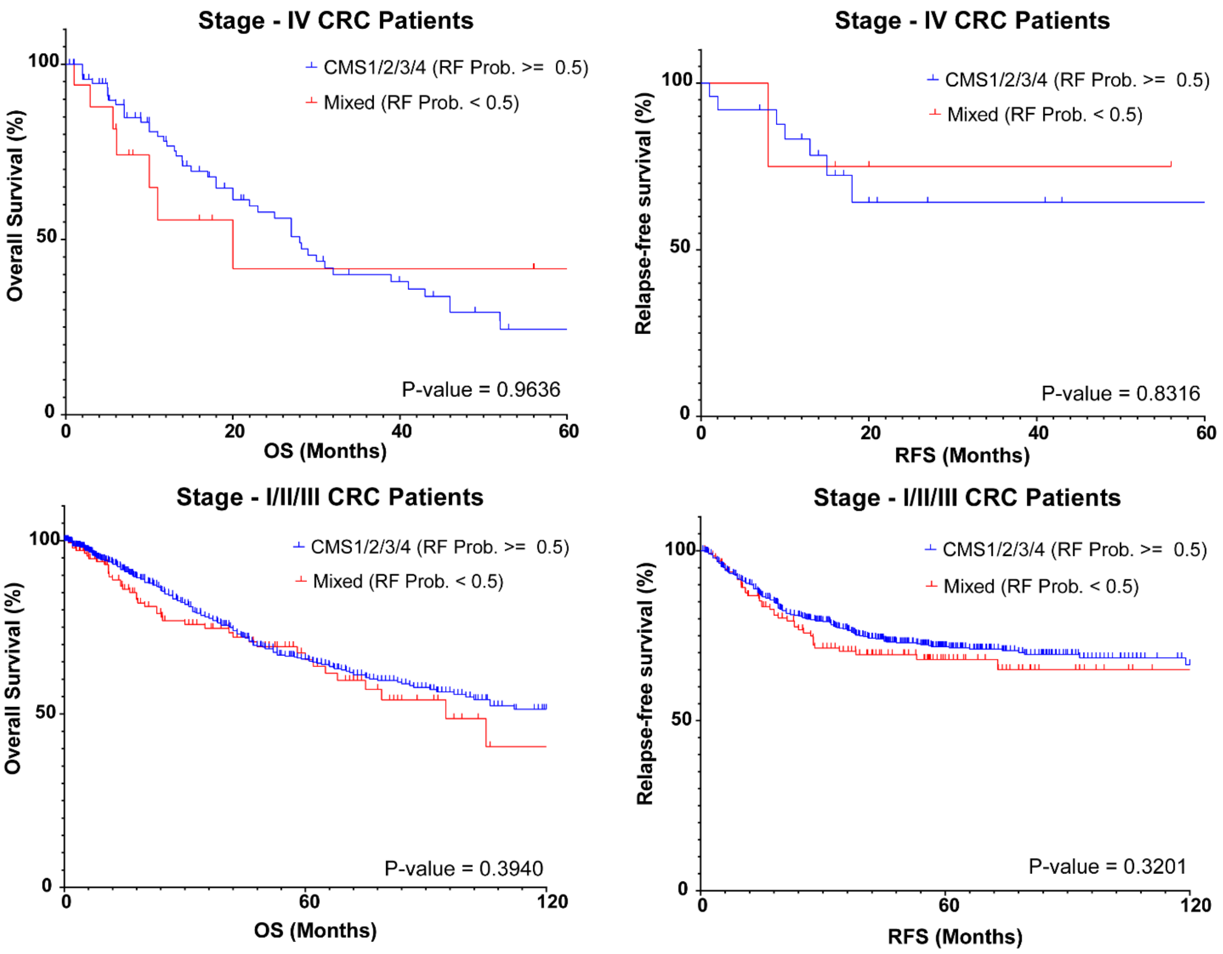

Figure S4: UMAP plot of discovery CRC single-cell dataset. (a) Same UMAP projection as Figure 4b,c but now colored by tumor sample (-B is border of tumor, -T is core of tumor). Note that there is grouping by sample in the epithelial cells only (not stromal or immune cells), as is expected given inter-tumor heterogeneity in gene expression. **(b)** Single-cell transcriptomic expressions of immune cells markers PD1, PD-L1, FOXP3, and CD163 in discovery dataset.

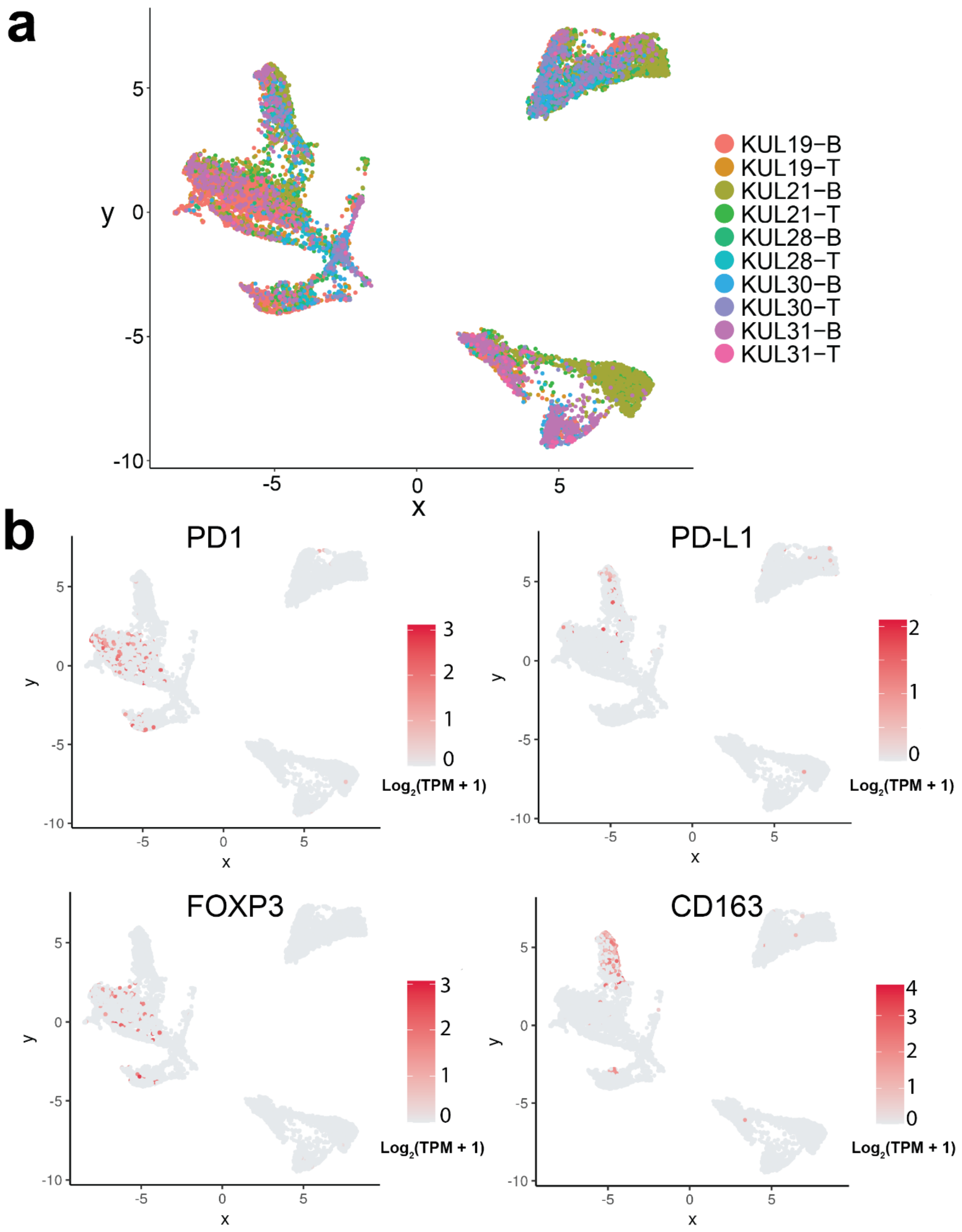

Figure S5: UMAP plots of specific CMS signal in the discovery dataset. Same UMAP projection as Figure 4b,c but now colored by scCMS scores of invidual cells. Note that scCMS scores (Minimum: 0 and Maximum: 10) of individual cells were converted into the percentage scale in UMAP color bars.

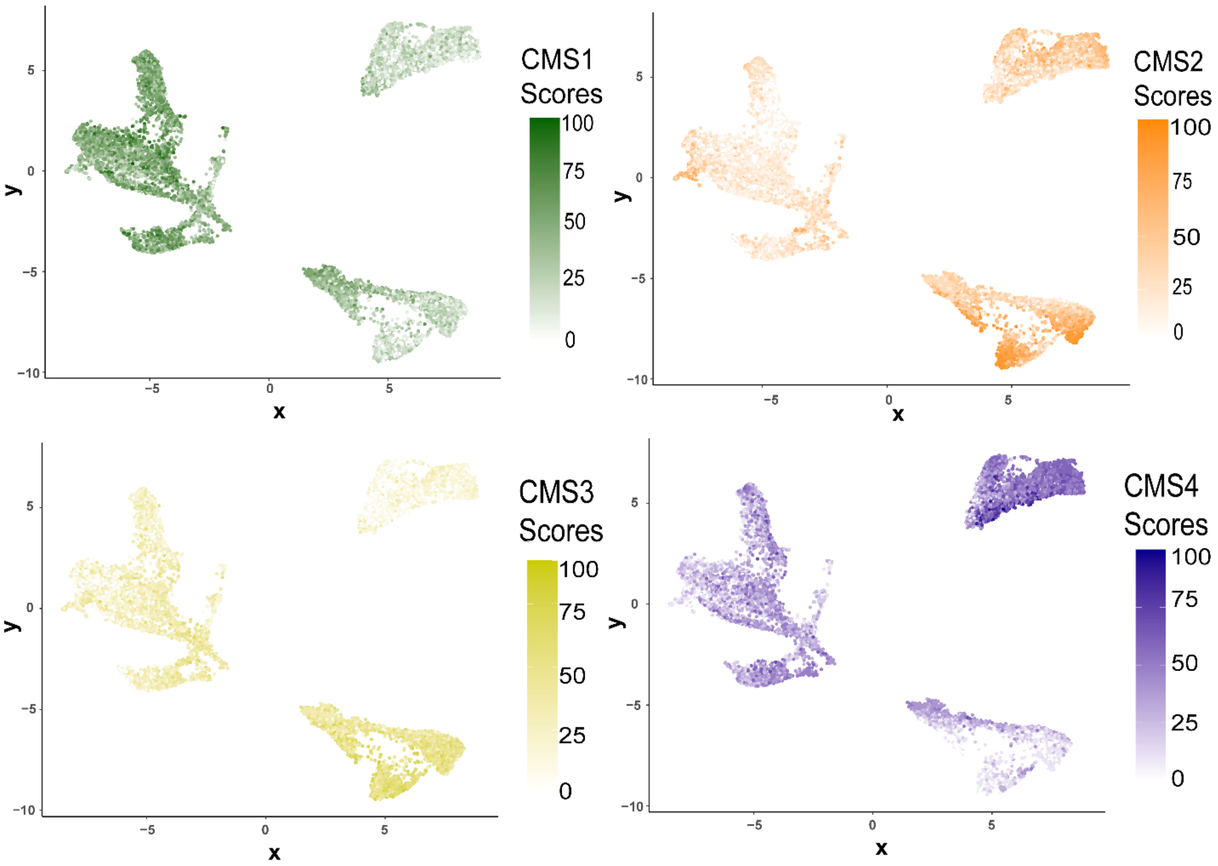

Supplement: Supplementary file 1 [file cancers-13-04923-s001.zip › cancers-1378038-supplementary.pdf]
